# Regulatory coordination of clustered microRNAs based on microRNA-transcription factor regulatory network

**DOI:** 10.1186/1752-0509-5-199

**Published:** 2011-12-16

**Authors:** Jin Wang, Martin Haubrock, Kun-Ming Cao, Xu Hua, Chen-Yu Zhang, Edgar Wingender, Jie Li

**Affiliations:** 1 The State Key Laboratory of Pharmaceutical Biotechnology and Jiangsu Engineering Research Center for MicroRNA Biology and Biotechnology; 2 Department of Bioinformatics, Medical School, Georg August University of Göttingen, Goldschmidtstrasse 1, D-37077 Göttingen, Germany

## Abstract

**Background:**

MicroRNA (miRNA) is a class of small RNAs of ~22nt which play essential roles in many crucial biological processes and numerous human diseases at post-transcriptional level of gene expression. It has been revealed that miRNA genes tend to be clustered, and the miRNAs organized into one cluster are usually transcribed coordinately. This implies a coordinated regulation mode exerted by clustered miRNAs. However, how the clustered miRNAs coordinate their regulations on large scale gene expression is still unclear.

**Results:**

We constructed the miRNA-transcription factor regulatory network that contains the interactions between transcription factors (TFs), miRNAs and non-TF protein-coding genes, and made a genome-wide study on the regulatory coordination of clustered miRNAs. We found that there are two types of miRNA clusters, i.e. homo-clusters that contain miRNAs of the same family and hetero-clusters that contain miRNAs of various families. In general, the homo-clustered as well as the hetero-clustered miRNAs both exhibit coordinated regulation since the miRNAs belonging to one cluster tend to be involved in the same network module, which performs a relatively isolated biological function. However, the homo-clustered miRNAs show a direct regulatory coordination that is realized by one-step regulation (i.e. the direct regulation of the coordinated targets), whereas the hetero-clustered miRNAs show an indirect regulatory coordination that is realized by a regulation comprising at least three steps (e.g. the regulation on the coordinated targets by a miRNA through a sequential action of two TFs). The direct and indirect regulation target different categories of genes, the former predominantly regulating genes involved in emergent responses, the latter targeting genes that imply long-term effects.

**Conclusion:**

The genomic clustering of miRNAs is closely related to the coordinated regulation in the gene regulatory network. The pattern of regulatory coordination is dependent on the composition of the miRNA cluster. The homo-clustered miRNAs mainly coordinate their regulation rapidly, while the hetero-clustered miRNAs exert control with a delay. The diverse pattern of regulatory coordination suggests distinct roles of the homo-clustered and the hetero-clustered miRNAs in biological processes.

## Background

MicroRNA (miRNA) is a class of ~22 nt small non-coding RNAs (ncRNAs) which inhibit gene expression at post-transcriptional stage by binding to the 3'UTR of mRNAs. They play essential roles in many crucial biological processes, including development, differentiation, apoptosis and cell proliferation [1-4], as well as numerous human diseases, such as chronic lymphocytic leukemia, fragile X syndrome, and various types of cancers [5-8].

The studies on the biogenesis of miRNAs [9,10] show that miRNA is firstly transcribed as pri-miRNA (i.e. primary miRNA) in the nucleus, then exported to the cytoplasm after being cleaved into pre-miRNA (precursor miRNA). In cytoplasm, pre-miRNA is processed into mature miRNA and incorporated into the RNA-induced silencing complex (RISC) which subsequently binds to the 3'UTR of mRNAs. The conversion of pre-miRNA to mature miRNA is generally one-to-one (i.e. one pre-miRNA once generates one mature miRNA), although both strands of pre-miRNAs could potentially become mature miRNAs [11]. However, the splicing of one pri-miRNA could result in multiple pre-miRNAs. This is because miRNA genes tend to be clustered in the genome [12] and one pri-miRNA could be a cluster of several miRNAs [13].

A set of miRNAs that are closely distributed in genome is termed as the miRNA cluster. The clustering propensity of miRNAs was first been discovered by large-scale surveys of small ncRNAs [14,15]. At present, it has been confirmed that miRNA clusters are widely distributed in animal genomes [16,17]. The conservation of miRNA clusters across species [18] indicates that miRNA clusters adapt special regulatory functions in biological processes. In addition, it has been shown by expression studies that the clustered miRNAs are often co-expressed [19-21], suggesting that they are jointly transcribed as a polycistron. Thus, the hypothesis comes up that the genomic coordination of clustered miRNA genes, which further leads to their coordinated transcription, will consequently result in a functional coordination. However, it is still unclear how the clustered miRNAs function coordinately.

Recently, Yuan X-Y *et al. *studied the functional coordination of clustered miRNAs based on the protein-protein interaction (PPI) network [22]. They found that the clustered miRNAs tend to target the mRNA of proteins that are located in the same functional module. While this kind of correlation supports the view of functional coordination of clustered miRNAs, little is known about the underlying mechanisms. In addition, the PPI network which is composed of direct protein-protein interactions cannot provide the successive regulation details of miRNAs. For example, the successive regulation on a protein by a miRNA through one or more TFs is not included in the PPI network.

Here, we studied the regulatory coordination of clustered miRNAs based on the miRNA-Transcription factor (miRNA-TF) regulatory network that comprises the interactions between transcription factors (TF), miRNAs and non-TF protein-coding genes. We found that there are two types of miRNA clusters, i.e. homo-clusters (miRNA clusters composed of miRNAs from a single miRNA family) and hetero-clusters (miRNA clusters composed of miRNAs from multiple miRNA families). In general, both the homo- and the hetero-clustered miRNAs show the behavior of regulatory coordination. However, the ways of regulatory coordination of both types of clustered miRNAs are different. The homo-clustered miRNAs show a direct regulatory coordination which is realized by a single regulation step (i.e. direct regulation), and tend to be involved in emergency processes, whereas the hetero-clustered miRNAs show an indirect regulatory coordination which is accomplished by 3 or more steps, and tend to participate in more complex processes.

## Results

### Homologous and heterologous miRNA clusters

**Figure 1:**
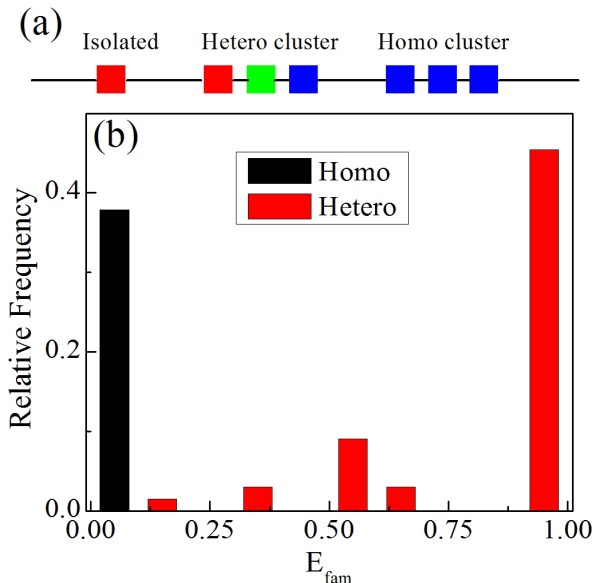
**Homo-clusters and hetero-clusters of miRNA**. a) Illustration of miRNA distributions in genome. The miRNAs of the same family are represented as squares of the same color; b) family entropy distribution of miRNA clusters.

There are in total 66 miRNA clusters of human (see Methods), which can be classified into two types. One is the homologous cluster (homo-cluster) composed of several miRNAs from the same family, and the other is the heterologous cluster (hetero-cluster) composed of miRNAs of various families (see Figure 1a and Additional File 1). We found that there are 25 homo-clusters and 41 hetero-clusters. The detailed family diversity of the miRNA clusters is characterized by the family entropy (*E_fam_*, see Methods). It is seen that the distribution of *E_fam _*shows a polarized behavior (see Figure 1b). Most of the family entropies are either 0 or 1. This means most of the 66 miRNA clusters are composed of the miRNAs from either one family or completely different families, suggesting that there is a fundamental difference of family composition between the homo-clusters and the hetero-clusters.

Usually, the miRNAs of the same family target a similar set of mRNAs, since they have the same seed regions [23] which predominantly determine the targets of miRNA [24]. Thus, it is suggested that the homo-clustered and the hetero-clustered miRNAs may have quite different regulatory features.

### Clustered miRNAs in network modules

In the miRNA-TF regulatory network containing the interactions between TF, miRNA and non-TF protein-coding genes (see Methods), 39 modules that have dense interactions were found using MCODE plus [25] of the network tool Cytoscape [26]. These modules altogether contain 47 homo-clustered miRNAs, 132 hetero-clustered miRNAs and 232 isolated miRNAs that are far from other miRNAs and do not belong to any clusters. The statistical analysis (see Methods) shows that the homo-clustered miRNAs and the hetero-clustered miRNAs are both significantly enriched (P < 0.01) in these 39 modules. Moreover, more than 50% of the modules (i.e. 22 out of 39) contain at least one miRNA pair that comes from the same miRNA cluster. The average cluster entropy (see Methods) of the 39 modules which describes the diversity of the miRNA clusters within a module is significantly lower (P < 0.01) than that in the random case in which miRNAs were randomly assigned to groups of the same number as human miRNA clusters (see Methods). These findings suggest that the miRNAs located within one genomic cluster tend to be involved preferentially in the same module. Such preference means that, in general, the clustered miRNAs exert a coordinated regulation since a module in a biological network usually represents a special or relatively independent biological function [27].

**Figure 2:**
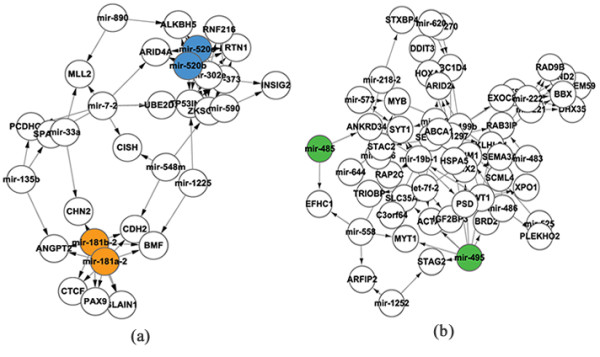
**Clustered miRNAs in two examples of modules**. a) A module containing homologous miRNAs from a homo-cluster (orange) and a hetero-cluster (blue); b) a module involving heterologous miRNAs from a hetero-cluster (green).

The preference of homo-clustered miRNAs in the same module is not surprising. Since the miRNAs in a homo-cluster bind to similar targets, they tend to form a local community of dense interactions associating them with common targets (see the orange circles in Figure 2a). Nevertheless, not all the homo-clustered miRNAs are involved in modules. The reason is that some homo-clustered miRNAs have such a small number of targets that the local community composed of these miRNAs and their targets is not dense enough to be included in a module. In addition, some homologous members of hetero-clustered miRNAs are found to be involved in the same modules (see the blue circles in Figure 2a). It seems that these homologous members of hetero-clustered miRNAs have the same type of coordinated regulation with that of the homo-clustered miRNAs. However, there are about 10% hetero-clusters in which the heterologous miRNA members appear in the same modules (see the green circles in Figure 2b). Since the coordinated regulation by these heterologous miRNAs obviously does not work through similar target sequences, it is assumed that there are distinct mechanisms underlying the coordinated regulation by hetero- and homo-clustered miRNAs.

### Distinctive regulatory coordination of homo- and hetero-clusters

To measure the regulatory coordination of clustered miRNAs quantitatively, we studied the target overlap of miRNA pairs in the same cluster. The target overlap (*TO*) of miRNA *i *and miRNA *j *is defined as:

(1) $$TO_{ij}=\frac{\overset{N}{\underset{k}{\sum }}S_{ik}S_{jk}}{\min\left(l_{i},l_{j}\right)+1}$$

where *l_i _*is the number of targets that are regulated by miRNA *i*, *N *is the total number of targets, and *s_ik _*is the element of the connecting matrix. *s_ik _*equals to 1 when miRNA *i *regulates target *k *directly (or indirectly), otherwise it equals to 0. Clearly, *TO_ij _*varies in the range of 0 to 1. The closer *TO_ij _*is to 1, the more targets miRNA *i *and miRNA *j *share, and the stronger is the coordinated regulation by miRNA *i *and miRNA *j*.

**Figure 3:**
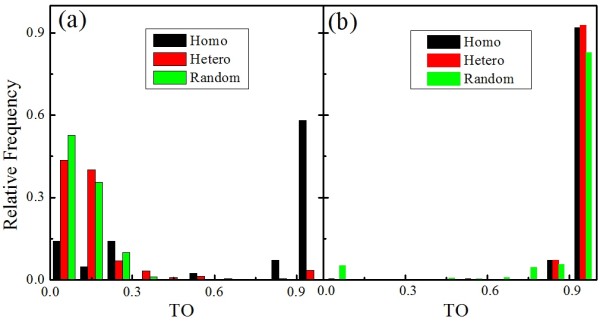
**Distribution of target overlaps for homo-clustered, hetero-clustered and random-clustered miRNAs**. a) Targets under direct regulation only; b) targets including indirect regulation.

We first studied the *TO *distribution by checking the direct targets of miRNAs. As expected, the homo-clustered miRNA pairs have high *TOs*. More than 75% miRNA pairs in homo-clusters are of the *TO*s higher than 0.8 (see Figure 3a). The average *TO *of homo-clusters (0.70) is significantly higher (P < 0.05) than those for hetero-clusters (0.15) and random clusters (0.10). Nevertheless, the *TOs *of the hetero-clustered miRNA pairs are as low as those of the miRNA pairs in random clusters (i.e. the clusters that are randomly generated by keeping the total number of miRNAs in each cluster, see Methods). About 90% miRNA pairs in hetero-clusters and random clusters are of the *TO*s lower than 0.3. This indicates that there is no regulatory coordination of hetero-clustered miRNAs within one step. However, the *TO *features of hetero-clustered miRNA pairs change when the indirect targets of miRNAs are additionally considered. The behavior of *TO *distribution for hetero-clusters looks more like that for the homo-clusters than the random clusters. Specifically, the relative frequency of hetero-clusters is apparently higher than that for random clusters when *TO *> 0.8 (see Figure 3b). The average *TO*s of homo-clusters and hetero-clusters which are 0.97 and 0.96 respectively are both significantly higher than that of random clusters as 0.83 (P < 0.05). This indicates that the regulatory coordination of the hetero-clustered miRNAs comes up by indirect regulations.

**Figure 4:**
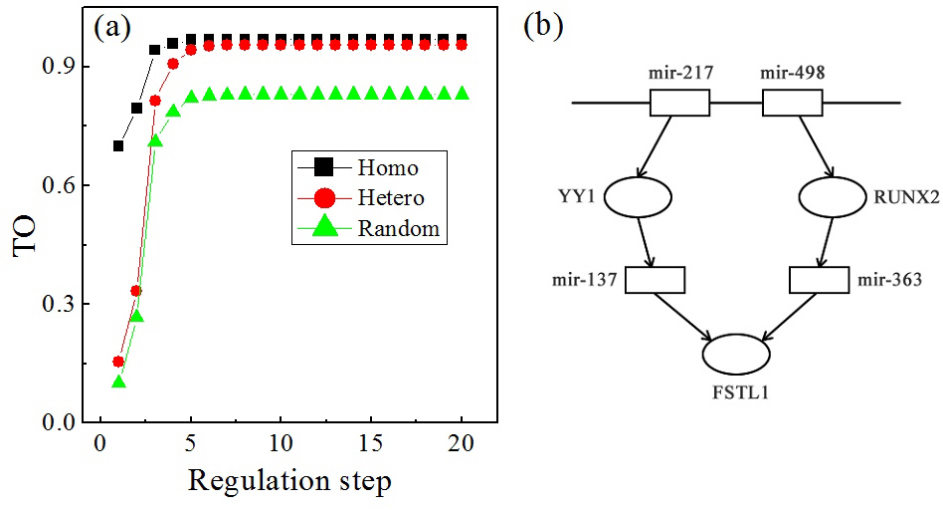
**regulatory coordination by regulation steps**. a) Variation of target overlap (TO) with the number of regulation steps; b) illustration of 3-step regulations.

Furthermore, we analyzed the dependence of the average *TO *on the number of regulation steps for homo-clusters, hetero-clusters and random clusters. A regulation step equal to 1 means that all the considered targets are directly regulated by miRNAs. As shown in Figure 4, the *TO*s of homo-clusters and hetero-clusters, which are both consistently higher than those of the random clusters, saturate when the regulation steps is more than 5. However, there are distinct differences between the curves of homo-clusters and hetero-clusters. While the *TO *curve of hetero-clusters is first close to that of random clusters for regulation steps less than 3, it approaches the curve of homo-clusters and become significantly higher than that of random clusters (P < 0.05) as the regulation step is larger than 3. This means that the regulatory coordination of hetero-clustered miRNAs occurs after 3 steps (see example in Figure 4b), unlike that of homo-clustered miRNAs, where it is already effective at the first step (i.e. the direct regulation). Such distinction between regulatory coordination mechanisms may indicate distinct roles of the homo- and hetero-clustered miRNAs in biological processes.

### Functional analysis of targets of homo- and hetero-clusters

The function of miRNA targets was analyzed using the tool DAVID (http://david.abcc.ncifcrf.gov/, see Methods). We found that the targets of homo-clustered miRNAs are significantly involved in emergency processes that need to be preceded rapidly, such as response to stimuli and the intrinsic apoptotic pathway that involves mitochondria. The latter represents an emergency process since the intrinsic apoptotic pathway is usually activated to induce a rapid cell death [28]. In contrast, the hetero-clustered miRNAs are involved in complex biological processes including the metabolism and the extrinsic apoptotic pathway that happens primarily in the cytoplasm. These processes are generally of less urgency, but they are more complex than those of homo-clusters. It has been revealed that thousands of genes and reactions are involved in metabolic processes [29], and the extrinsic apoptotic pathway is composed of several complex caspase processes [28].

Moreover, the extrinsic apoptotic process is also more complex than the internal apoptotic process since it comprises significantly more reactions [28]. Besides, three functions (i.e. signal transduction, development and transport) are shared targets of homo- and hetero-clustered miRNAs.

## Discussion

MicroRNA clusters, which are groups of tandem miRNA genes that are closely located in the genome, are abundantly and widely distributed in animal genomes. It has been revealed that about 50% of the miRNA genes in *Drosophila *[15] and over 30% of the miRNA genes in human, mouse, rat and chicken are located in clusters [30]. Co-expression experiments of clustered miRNA genes show that one miRNA cluster is usually transcribed as a single transcriptional unit [21]. This suggests the existence of some kind of regulatory coordination between the clustered miRNAs. However, it is still unclear how the clustered miRNAs coordinate their regulation. Here, we describe a genome-wide study on the regulatory coordination of clustered miRNAs based on the miRNA-TF regulatory network.

The miRNA clusters are classified into homo-clusters that contain miRNAs of the same family and hetero-clusters that contain miRNAs of multiple families. Most of the miRNA clusters are either homo-clusters or hetero-clusters of miRNAs with completely different families. Such polarized behavior indicates that the family composition of clustered miRNAs may be an important characteristic that is closely related to the regulatory features of miRNA clusters.

In this study, we have used a miRNA-TF regulatory network that represents the regulation exerted by TFs and miRNAs on gene expression of target genes. This regulatory network presents a comprehensive view on the regulations of miRNAs since it involves the transcriptional regulation of miRNAs genes by TFs as well as the direct regulation of the targeted mRNAs by miRNAs. Such regulatory network has previously been used to study the combinatory regulation of miRNAs and TFs on gene expression. For example, different types of miRNA-TF co-regulations have been revealed based on the miRNA-TF regulatory network [31,32]. In addition, it is reported by Kang *et al. *that there are two-layer regulations on the gene expression, where TFs function as important mediators of miRNA-initiated regulatory effects [33]. These studies suggest that the miRNA-TF regulatory network is a good substrate for studying the complex regulatory features of miRNAs.

The result that the clustered miRNAs, whether they are from homo- or hetero-clusters, preferably exert their effect in one module suggests a general regulatory coordination of clustered miRNAs. Intuitively, the regulatory coordination of homo-clustered miRNAs is ascribed to the high sequence similarity of homologous miRNAs. However, not all the sequences of homologous miRNAs are similar enough to bind to the same targets. More than half of the homologous miRNA pairs share less than 50% of the targets (see Additional File 2). An example is miR-329 family, in which any pairs of the three members (i.e. hsa-mir-543, hsa-mir-329, hsa-mir-495) share less than 20% of the targets. The homo-clustered miRNAs are the homologous ones that share large amount of targets. This suggests that the homo-clustered miRNAs are not the arbitrary homologous miRNAs, but the ones finely designed for the regulatory coordination. The target overlaps of hetero-clustered miRNAs are much smaller than the homo-clustered miRNAs, but they similarly appear in the same modules. This indicates that the homo-clustered miRNAs and the hetero-clustered miRNAs have distinctive ways of coordinated regulation. In addition, there are some cases that not all the members of miRNAs in homo-/hetero-clusters are found in the same modules. One possible reason is that the size of modules depends on the parameter that scales the density of interactions. If the parameter is strict, the resulting modules, which are generally of small size, will include few miRNA cluster members. Another reason may be that 10 kb is not an accurate cutoff for the definition of miRNA clusters. The miRNA cluster members that are not found in the same modules may not be included anymore in the cluster when there is a little deviation of the cutoff.

It is clear that the regulatory coordination of homo-clustered miRNAs is achieved by one regulation step (i.e. at the level of direct targets of miRNAs), since the homologous miRNAs in homo-clusters have almost the same targets. However, the regulatory coordination of hetero-clustered miRNAs is realized by at least 3 steps. Thus, the regulatory coordination of homo-clustered miRNAs is direct, while that of hetero-clustered miRNAs is indirect. These two types of coordinated regulation both have their own advantage. Since there is no intermediate regulator between the miRNAs and their targets, the direct coordinated regulation has the advantage of accuracy and quickness. Whereas, the indirect coordinated regulation has the advantage of variety since diverse types of coordinated regulation can be realized by inducing additional cross-regulations between intermediated regulators (see Additional File 3).

**Figure 5:**
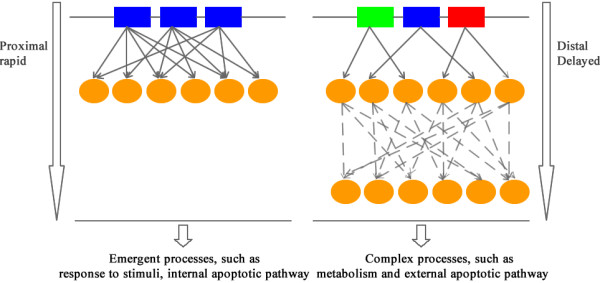
**Illustration of regulations for homo-clusters and hetero-clusters**. Homo-clustered miRNAs are represented as lined squares of a same color, and hetero-clustered miRNAs as lined squares of different colors. Eclipses are TF/non-TF genes. Solid arrows denote direct regulation, and dash arrows indirect regulation.

In general, the regulatory coordination of clustered miRNAs is to guarantee the validity and efficiency of miRNA regulations in a certain biological process. The direct regulatory coordination means that the effective regulation is a rapid one which is capable to cope with an emergency situation (see Figure 5). Thus, the homo-clusters could be involved in biological processes that match this requirement, such as response to certain stimuli. However, a rapid coordinated regulation pushing the affected system into a certain direction is accompanied by the risk to let the corresponding biological processes run out of control unless there are additional control processes counter-acting this push. Furthermore, the incorporation of more intermediates may increase the flexibility of regulation. Therefore, the indirect regulatory coordination that results in a delayed regulation may be adopted by complex biological processes such as metabolism.

## Conclusion

Our study is focused on the internal coordination of genomic-clustered miRNAs. The results suggest that there are two types of miRNA clusters, i.e. homo-clusters that contain miRNAs of the same family and hetero-clusters that contain miRNAs of multiple families. These two types of miRNA clusters show distinct behaviors of regulatory coordination in the gene regulatory network that represents the direct interactions between miRNA, TF and non-TF protein-coding genes. The homo-clusters show a direct regulatory coordination and tend to be involved in biological processes of emergency situations, while the hetero-clusters show an indirect regulatory coordination and tend to take part in more complex biological processes.

Our study shows the diversity of miRNA regulations responding to the complex requirements of biological functions and contributes to understand the complex function and regulatory mechanism of miRNAs at a network scale.

## Methods

### miRNA clusters

All the human miRNAs along with their genomic information were retrieved from miRBase 13.0 [34]. Two miRNAs that are consecutively located within 10 kb of each other were considered to belong to one miRNA cluster. This definition about miRNA clusters is based on the study of miRNA genomic distribution. In previous, it has been revealed that the distances between miRNA pairs located consecutively in genome are following a biomodal distribution. The valley between the two peaks is located at around 10 kb, suggesting that 10 kb may be the reasonable cutoff to define miRNA clusters [31]. In total, there are 718 human precursor miRNAs (pre-miRNAs) of distinct genomic locations. Among these 718 pre-miRNAs, about 36% form 66 miRNA clusters. The number of miRNA clusters is stable as the cutoff deviates from 10 kb. It varies at the range of 64-67 as the cutoff increases from 5 kb to 15 kb. The miRNAs in the same miRNA cluster composed of intronic/extronic miRNAs are of the same host genes (see Additional File 4), suggesting that the miRNA clusters tend to be transcribed into one transcript. For the intergenic miRNA clusters, it is suggested that the intergenic miRNA cluster may have short transcript less than 4 kbp [35]. This means our definition about intergenic miRNA clusters has the risk to draw an inaccurate conclusions since some of our intergenic miRNA clusters (about 30%) are much longer than 4 kbp. To detect whether our definition about intergenic miRNA clusters take a bias impact on the conclusion, we compared the coordinated behavior of our intergenic miRNA clusters with that of a newly-defined clusters which follow the constraint that the length of every intergenic miRNA cluster should be less than 4 kbp (i.e. the 4 kbp-constraint intergenic miRNA cluster). The results show that the direct and indirect coordinated behaviors of these two types of intergenic clusters are both very similar (see Additional File 5 and 6 for details). This suggests our definition on intergenic miRNA clusters is good enough for our study, and it does not take any bias impact on the final conclusion.

### MiRNA families

The information on miRNA families has been derived from Rfam database, in which various kinds of RNAs are classified into families based on their sequence and structural alignment [36]. A miRNA family generally means a collection of miRNAs that are derived from a common ancestor.

### Family entropy of a miRNA cluster

The family entropy, which is defined to characterize the diversity of miRNA families in a miRNA cluster, is defined as follows:

(2) $$E_{fam}=-\frac{1}{\ln N_{fam}}\overset{N_{fam}}{\underset{i}{\sum }}p_{i}\ln p_{i}$$

Given that a cluster comprises miRNAs of *N_fam _*families, *p_i _*is the probability that any miRNA in the cluster belongs to family *i*. *E_fam _*varies in the range of 0-1. *E_fam _*= 0 means that all the miRNAs in the cluster come from the same family, and *E_fam _*= 1 means that all clustered miRNAs come from different families.

### Construction of the regulatory network

To study the regulatory feature of the clustered miRNAs, we constructed the miRNA-TF regulatory network, which contains the regulatory relationships between TFs, miRNAs and non-TF protein-coding genes for human (see Additional File 7). Generally, there are two kinds of interactions in the network which respectively start from TFs and miRNAs. An interaction starting from a TF means that the TF regulates the transcription of the target, while an interaction starting from a miRNA means that the miRNA represses the translation of the target. We predicted the interactions starting from a TF by searching the conserved TF binding sites (TFBSs) within a putative promoter area 1 kb upstream the transcriptional start site of the target. Firstly, all the potential human TFBSs are collected from TRANSFAC (version 2009.4) [37] based on the position weighted matrix (PWM). Secondly, the conserved TFBSs are derived from the conserved promoter area across the 5 species of human, mouse, dog, cow and opossum based on RefSeq annotation of UCSC hg18 http://genome.ucsc.edu/index.html. Finally, the conserved relationships between TFBSs and TFs are predicted using the Match-algorithm provided by TRANSFAC. Note that the prediction of the TFBSs is done based on phylogenetic footprinting from five mammalian genomes in order to limit the false positive of the TFBS prediction. Since it has been discussed that the miRNAs within the same cluster are often transcribed simultaneously, we took a whole cluster of miRNAs as a single unit, and searched the TFBSs in the 10 kb upstream to the start point of the first miRNA in the cluster. In addition, we predicted the interactions starting from miRNAs by the three tools of Targetscan [24], Pictar [38] and Tarbase [39]. The union results predicted by these three tools are taken to give a comprehensive regulatory network so that many potential instants of regulatory coordination will be dug out to help further studies in experiment. Replacing the union results with the results of Tarbase which are verified by experiments does not affect our conclusion.

### Statistic analysis on the clustered miRNAs in modules

#### Enrichment of homo-/hetero- clusters

The enrichment of homo-/hetero-clusters in the 39 modules is evaluated based on the random distribution of the average homo-/hetero-clustered miRNA numbers in modules. Firstly, 1000 random cases are generated by randomly re-participating miRNAs into the 39 modules with the total miRNA number in each module kept. Then the random distribution is obtained by calculating the average homo-/hetero-clustered miRNA numbers in modules. Finally, the significance of the enrichment of homo-/hetero-clustered miRNAs in modules is evaluated based on the random distribution.

#### miRNA cluster entropy of a module

The miRNA cluster entropy of a module (*E_c_*) is defined as follows:

(3) $$E_{c}=-\frac{1}{\ln N_{c}}\overset{N_{c}}{\underset{i}{\sum }}{p^{c}}_{i}\ln{p^{c}}_{i}$$

Note that *p^c^_i _*is the probability of miRNA cluster *i *occurring in a module and *N_c _*is the total number of miRNA clusters in the module. Similar as *E_fam_*, *E_c _*varies at the range of 0-1. *E_c _*= 0 means that all the miRNAs in the module are in a same cluster, and *E_c _*= 1 means that the miRNAs are from completely different clusters.

### Random clusters of miRNAs

There are totally 458 isolated miRNAs and 66 miRNA clusters in genome. If the isolated miRNAs are considered as the pseudo-cluster, there are 524 (i.e. 458 + 66) clusters of miRNAs. Thus, a set of random clusters are generated by re-assigning miRNAs into these 524 clusters with the number of miRNAs in each cluster maintained. All the distributions for the random miRNA clusters are the ones averaged over 1000 random sets.

### Functional analysis of miRNA targets

The public tool DAVID is used to analyze the functions of the targets of homo-clustered and hetero-clustered miRNAs. GO [40] is selected as the annotated database. The annotated level is set at "GOTERM_BP_5". "Functional Annotation Clustering" with default classification stringency is applied to derive all the related functions associating with their enrichment scores and P-values. The enriched functions are defined as the ones with P-value less than 0.01 and enrichment score more than 1.0 [41]. All of the enriched functions for the targets of homo-clustered and hetero-clustered miRNAs are respectively listed in Additional File 8 and 9. To make the feature of enriched functions more clear, we manually curated and re-categorized the enriched functions as shown in Table 1 (Details in Additional File 8 and 9). Each enrichment score and P-value for a functional category are the average on the values of all the included functions.

**Table 1:** Enriched functions of miRNA targets.

Homo-cluster			Hetero-cluster		
**Functional categories**	**Enrichment score**	**P-value**	**Functional categories**	**Enrichment score**	**P-value**

Response to stimuli	1.73	0.004	Metabolism	2.60	0.002

Internal apoptotic pathway	1.64	0.002	External apoptotic pathway	2.08	0.003

Signal transduction	1.43	0.008	Signal transduction	2.20	0.005

Development	1.37	0.004	Development	1.50	0.007

Transport	1.21	0.007	Transport	1.25	0.005

The enriched function, enrichment scores and P-values are given. The left three columns are for the targets of homo-clustered miRNAs, and the right three columns are for the targets of hetero-clustered miRNAs.

## Authors' contributions

JW did the network analysis on the regulatory coordination of clustered miRNAs. MH, KMC and XH participated in the construction of the miRNA-TF regulatory network. CYZ, EW and JL conceptualized and designed the study, coordinated among research collaborators, and helped to draft the manuscript. All authors read and approved the final manuscript.

## Supplementary Material

Additional file 1 **miRNA clusters**. All of the 66 miRNA clusters are listed associating with their types, i.e. homo-cluster or hetro-cluster. Click here for file

Additional file 2 **Distribution of target overlaps for homologous miRNAs**. The black is for the homologous miRNAs in homo-clusters, and the red for all the homologous miRNAs. Click here for file

Additional file 3 **Examples of 3-steps coordinated regulation of hetero-clustered miRNAs**. Six examples embedding the cross-regulation between intermediate regulators are illustrated. Click here for file

Additional file 4 **MiRNA clusters composed of intronic/extronic miRNAs**. The information about miRNA types and host genes of intronic/extronic clustered miRNAs is derived from miRBase 13.0. Click here for file

Additional file 5 **Direct regulatory coordination for two types of intergenic miRNA clusters**. Target overlap (TO) distribution describing the direct regulatory coordination. a) for our intergenic miRNA clusters; b) for 4 kbp-constraint intergenic miRNA clusters. The black, red and blue plots are respectively for the homo-clustered, hetero-clustered and randomized miRNA pairs (i.e. the miRNA pairs randomly selected from different intergenic miRNA clusters) that are located in intergenic miRNA clusters. The TO distributions show a similar behavior in figure a) and b), suggesting that the definition of the intergenic miRNA clusters has no impact on the property of the direct regulatory coordination. Click here for file

Additional file 6 **Indirect regulatory coordination for two types of intergenic miRNA clusters**. TO distribution describing the indirect regulatory coordination. a) for our intergenic miRNA clusters; b) for 4 kbp-constraint intergenic miRNA clusters. The black, red and blue plots are respectively for the homo-clustered, hetero-clustered and randomized miRNA pairs that are located in intergenic miRNA clusters. The TO distributions show a similar behavior in figure a) and b), suggesting that the definition of the intergenic miRNA clusters has no impact on the property of the indirect regulatory coordination. Click here for file

Additional file 7 **Regulatory network**. The whole regulatory network is provided in SIF format. Click here for file

Additional file 8 **Enriched functions of targets of homo-clustered miRNAs**. Detailed information about the enriched functions of the targets of homo-clustered miRNAs is given. Click here for file

Additional file 9 **Enriched functions of targets of hetero-clustered miRNAs**. Detailed information about the enriched functions of the targets of hetero-clustered miRNAs is given. Click here for file
