# Supplementary figures and images for: Regulatory coordination of clustered microRNAs based on microRNA-transcription factor regulatory network
Source: BMC Syst Biol. 2011 Dec 16;5:199. doi: 10.1186/1752-0509-5-199 (PMC3262773; doi:10.1186/1752-0509-5-199)

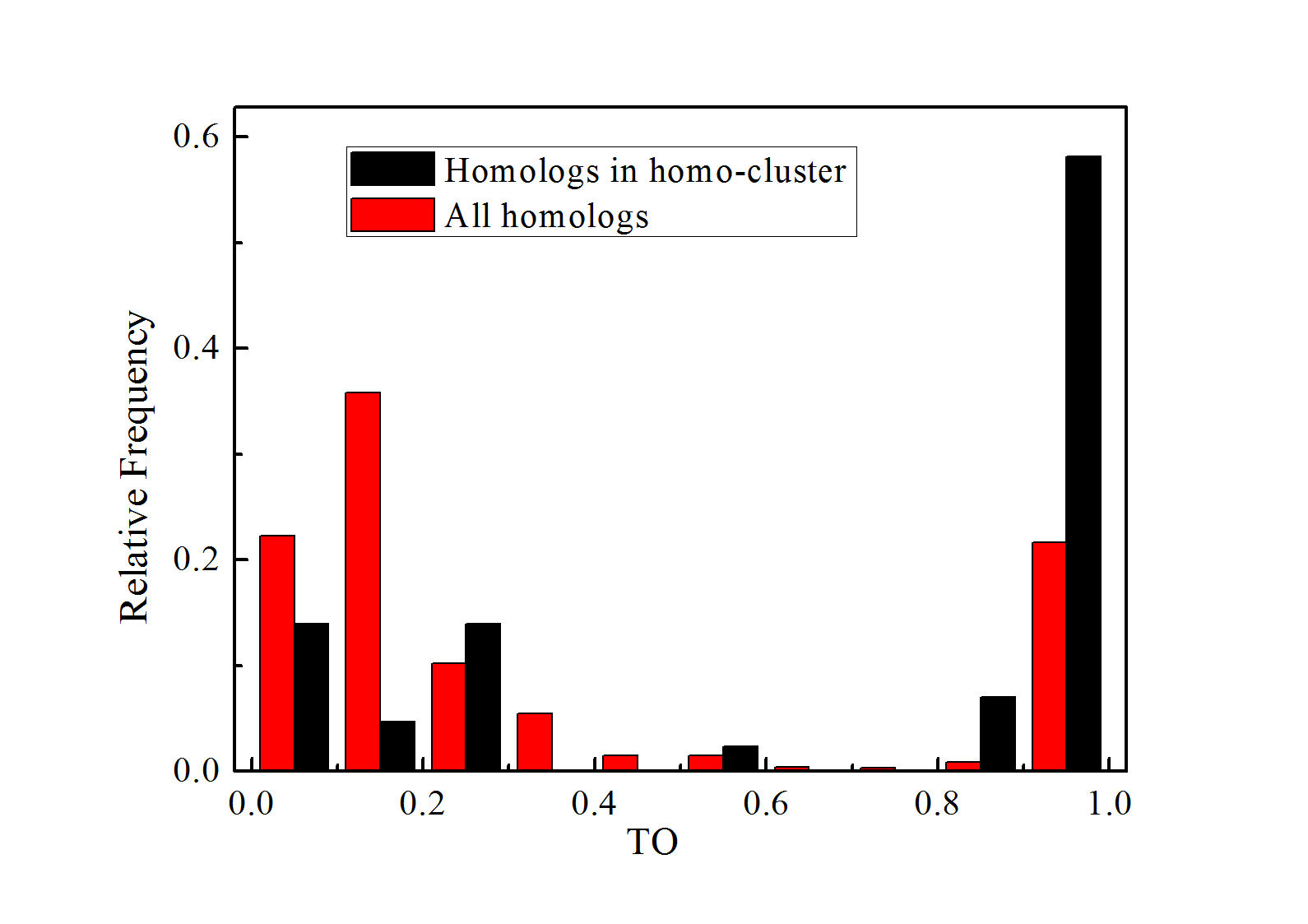

Supplement: Additional file 2 — Distribution of target overlaps for homologous miRNAs. The black is for the homologous miRNAs in homo-clusters, and the red for all the homologous miRNAs. [file 1752-0509-5-199-S2.JPEG]

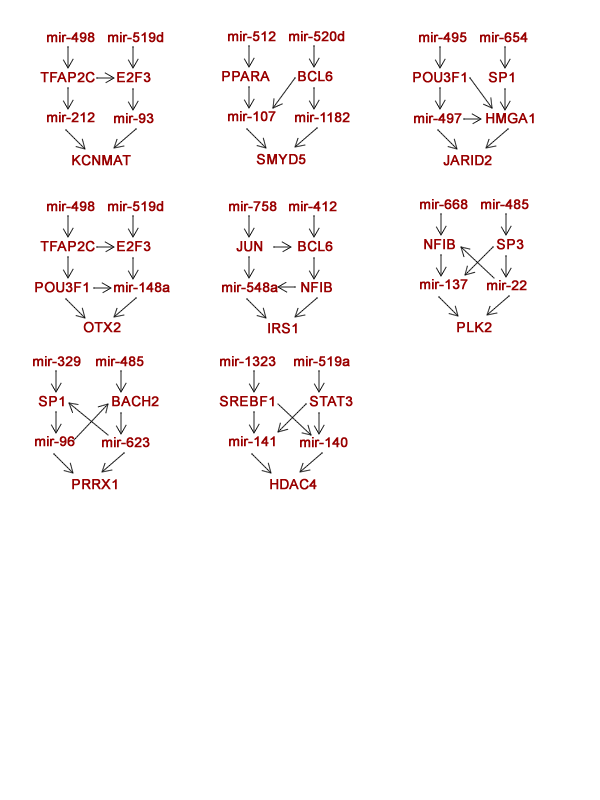

Supplement: Additional file 3 — Examples of 3-steps coordinated regulation of hetero-clustered miRNAs. Six examples embedding the cross-regulation between intermediate regulators are illustrated. [file 1752-0509-5-199-S3.PNG]

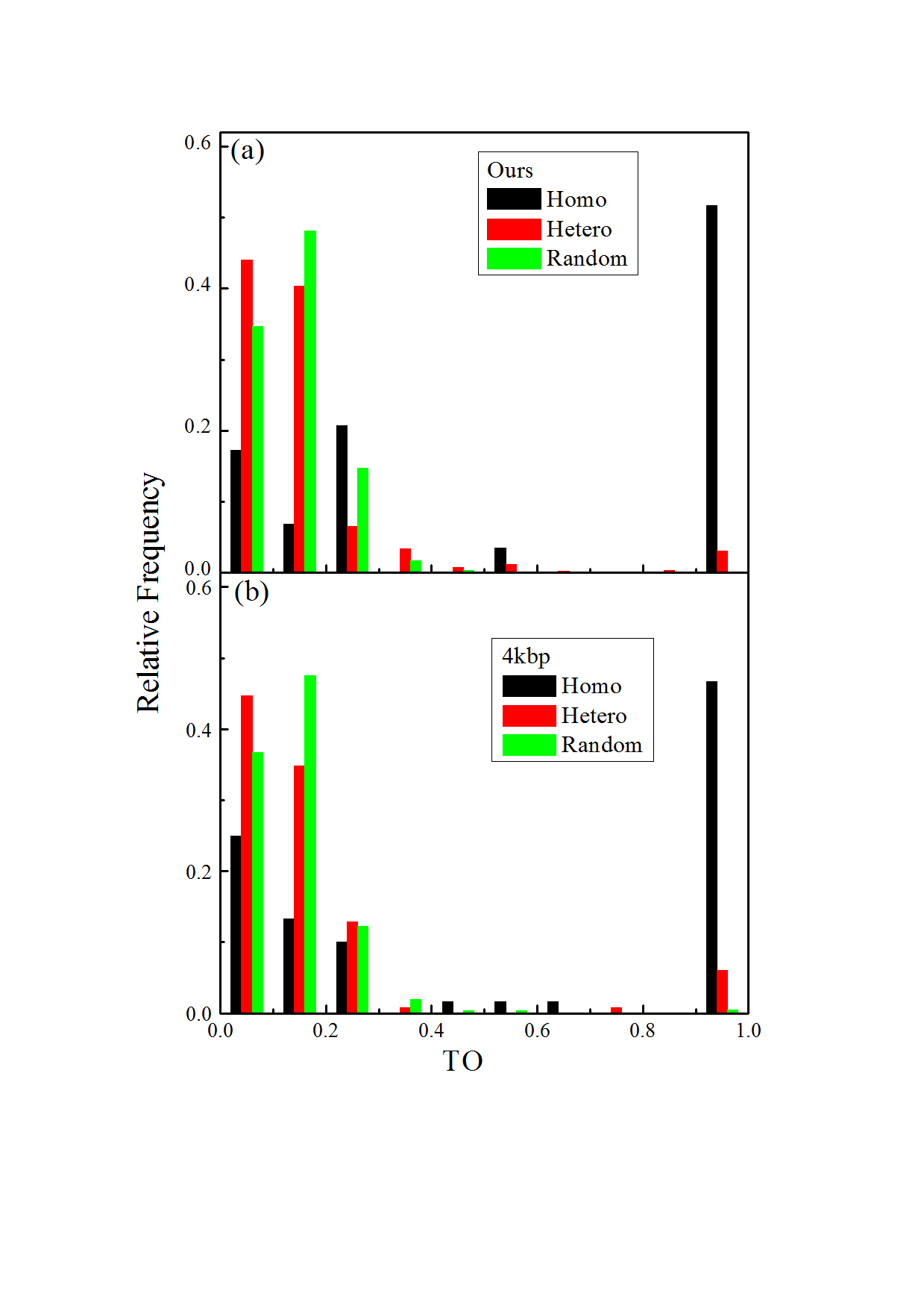

Supplement: Additional file 5 — Direct regulatory coordination for two types of intergenic miRNA clusters. Target overlap (TO) distribution describing the direct regulatory coordination. a) for our intergenic miRNA clusters; b) for 4 kbp-constraint intergenic miRNA clusters. The black, red and blue plots are respectively for the homo-clustered, hetero-clustered and randomized miRNA pairs (i.e. the miRNA pairs randomly selected from different intergenic miRNA clusters) that are located in intergenic miRNA clusters. The TO distributions show a similar behavior in figure a) and b), suggesting that the definition of the intergenic miRNA clusters has no impact on the property of the direct regulatory coordination. [file 1752-0509-5-199-S5.JPEG]

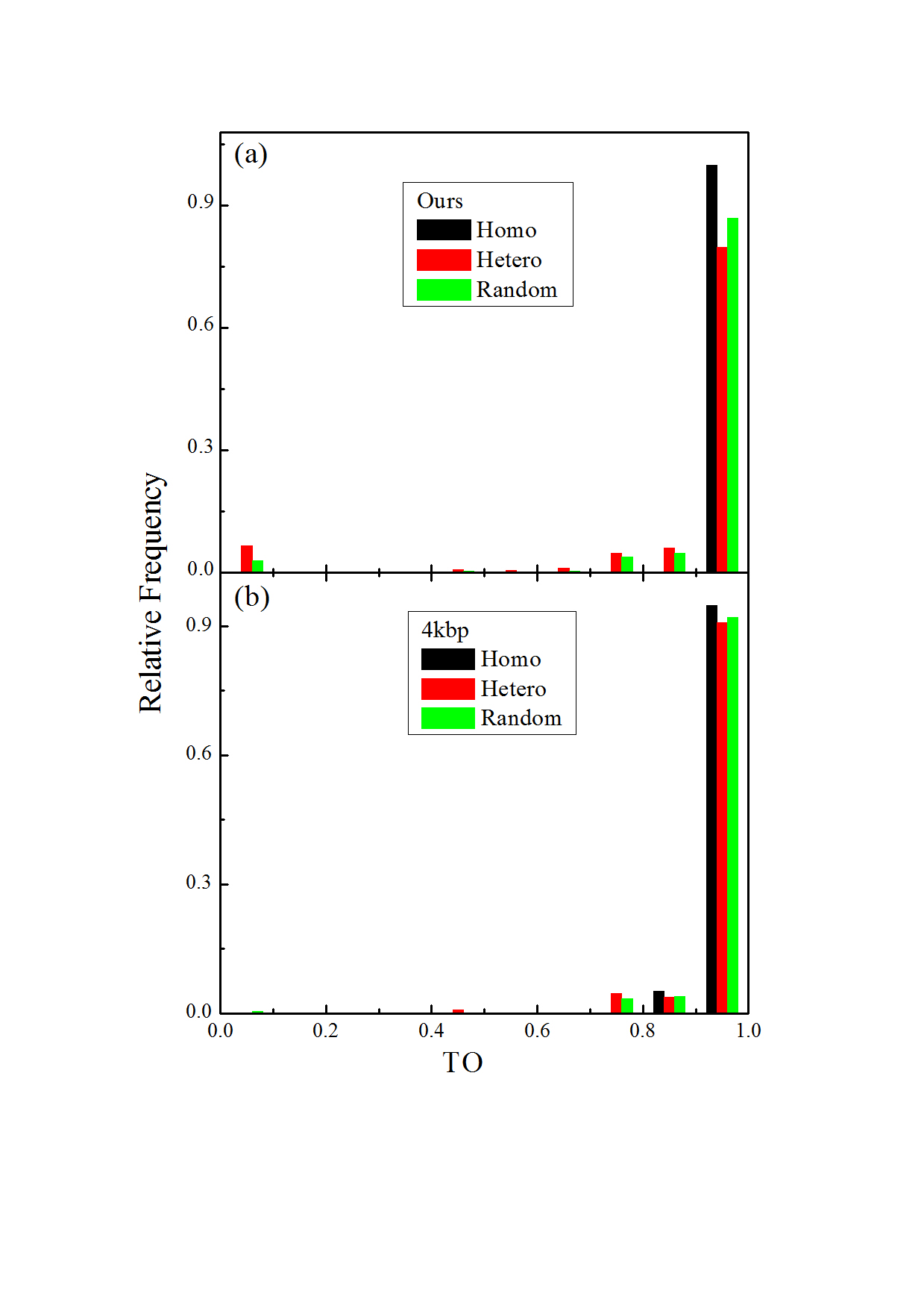

Supplement: Additional file 6 — Indirect regulatory coordination for two types of intergenic miRNA clusters. TO distribution describing the indirect regulatory coordination. a) for our intergenic miRNA clusters; b) for 4 kbp-constraint intergenic miRNA clusters. The black, red and blue plots are respectively for the homo-clustered, hetero-clustered and randomized miRNA pairs that are located in intergenic miRNA clusters. The TO distributions show a similar behavior in figure a) and b), suggesting that the definition of the intergenic miRNA clusters has no impact on the property of the indirect regulatory coordination. [file 1752-0509-5-199-S6.JPEG]
